# SARS-CoV-2 infection and transmission via the skin to oro-nasal route with the production of bioaerosols in the ferret model

**DOI:** 10.1099/jgv.0.002022

**Published:** 2024-09-18

**Authors:** Rebecca Shipley, Amanda H. Seekings, Alexander M.P. Byrne, Shweta Shukla, Joe James, Hooman Goharriz, Fabian Z.X. Lean, Alejandro Núñez, Anthony R. Fooks, Lorraine M. McElhinney, Sharon M. Brookes

**Affiliations:** 1 Virology Department, Animal and Plant Health Agency (APHA-Weybridge), Addlestone, Surrey, KT15 3NB, UK; 2 Pathology and Animal Sciences Department, Animal and Plant Health Agency (APHA-Weybridge), Addlestone, Surrey, KT15 3NB, UK; 3 Department of Pathobiology and Population Sciences, Royal Veterinary College, North Mymms, Hertfordshire, UK

**Keywords:** Alpha, Beta, bioaerosol, Delta, ferret, Gamma, SARS-CoV-2, skin transference, transmission, zoonoses

## Abstract

Direct and indirect transmission of severe acute respiratory syndrome coronavirus 2 (SARS-CoV-2) has been attributed to virus survival in droplets, bioaerosols and on fomites including skin and surfaces. Survival of SARS-CoV-2 variants of concern (Alpha, Beta, Gamma, and Delta) on the skin and virus transference following rounds of skin-to-skin contact were assessed on porcine skin as a surrogate for human skin. SARS-CoV-2 variants were detectable on skin by RT-qPCR after 72 h at biologically relevant temperatures (35.2 °C) with viral RNA (vRNA) detected after ten successive skin-to-skin contacts. Skin-to-skin virus transmission to establish infection in ferrets as a model for mild/asymptomatic SARS-CoV-2 infection in mustelids and humans was also investigated and compared to intranasal ferret inoculation. Naïve ferrets exposed to Delta variant SARS-CoV-2 in a ‘wet’ or ‘dry’ form on porcine skin resulted in robust infection with shedding detectable for up to 14 days post-exposure, at comparable viral loads to ferrets inoculated intranasally. Transmission of SARS-CoV-2 to naïve ferrets in direct contact with infected ferrets was achieved, with environmental contamination detected from ferret fur swabs and air samples. Genetic substitutions were identified in bioaerosol samples acquired following single contact passage in ferrets, including Spike, ORF1ab, and ORF3a protein sequences, suggesting a utility for monitoring host adaptation and virus evolution via air sampling. The longevity of SARS-CoV-2 variants survival directly on the skin and skin-to-skin transference, enabling subsequent infection via the skin to oro-nasal contact route, could represent a pathway for SARS-CoV-2 infection with implications to public and veterinary health.

## Introduction

Severe acute respiratory syndrome coronavirus 2 (SARS-CoV-2) was first detected in humans in 2019, in Wuhan, China [1]. Coronavirus disease 2019 (COVID-19) in humans is caused by SARS-CoV-2 infection and has been globally reported to have a case fatality rate of 2–3% [2]. Following widespread infection among humans, COVID-19 was officially declared a pandemic by the World Health Organization in March 2020 [3] and continues to circulate in the human population with seasonal peaks in prevalence [4].

The origin of the COVID-19 pandemic remains elusive although genetic evidence for infected animal hosts prior to detections in humans have been described [5]. Spillback of SARS-CoV-2 infection into animals has also been reported including mustelids (mink and ferrets) [6,8], cervids (white-tailed deer) [9,10], canids (domestic dogs) [11], felids (domestic cats, tigers, and lions) [11,13], rodents (hamsters) [14,15], and several other species that exist as isolated case reports [16]. In addition to natural infection, a wide range of species susceptible to SARS-CoV-2 infection have been identified through *in vivo* and *in vitro* experimental studies, including ferrets, cats, rabbits, hamsters, non-human primates, foxes, white-tailed deer and bats [6,11, 17,20].

Notable SARS-CoV-2 animal infections include infection in mink, as they represent the first animal species with sustained intraspecies transmission and secondary spillover potential. Several outbreaks were reported in mink farms in the US, Canada, and Europe, with the first outbreak reported in the Netherlands in 2020 [21,23]. In addition to initial infection and sustained animal-to-animal transmission, the subsequent generation of novel variants resulted in interspecies transmission back into humans. In Denmark, despite a mass cull of mink, a significant percentage of the strains infecting humans were mink-derived, suggestive of a secondary zoonotic transmission event [24,25]. Other notable spillovers include zoo animals, domestic pets, white-tailed deer, and hamsters, where onward intra- and inter-species transmission has been reported to have occurred [16]. With evidence of zoonotic and reverse-zoonotic transmission and broad host range of SARS-CoV-2, there is obvious potential for the establishment of an animal reservoir, as demonstrated in the white-tailed deer [26]. Consequently, SARS-CoV-2 infection in animals was made a reportable disease in March 2021 by the World Organisation for Animal Health (WOAH) [27].

In humans, secretions from the respiratory tract or excreta containing SARS-CoV-2 can facilitate SARS-CoV-2 transmission [28]. Viral infection is initiated when epithelial cells in the mucous membranes of a susceptible individual are exposed to SARS-CoV-2 virions and rapid spread could be indicative of direct or indirect transmission through both droplets and fomites [28]. Evidence for detectable SARS-CoV-2 in aerosols generated by COVID-19 patients [29,30] and experimental studies recovering infectious SARS-CoV-2 from air samples [31,32] support the hypothesis that SARS-CoV-2 can be transmitted by the airborne route. In limited experimental studies in ferrets, cats, and hamsters, it has been shown that SARS-CoV-2 can transmit via direct contact and through aerosols [14,33,36]. In addition, non-contact transmission is reduced among experimental animals when partitioned by surgical masks [37], indicative that the bioaerosol transmission route is critical. Conversely, it has been suggested that allogrooming or human-handling may facilitate transmission as SARS-CoV-2 RNA was detected on the fur of ferrets following intranasal inoculation [33,38, 39]. However, the stability of SARS-CoV-2 on fur and skin and the infectious dose necessary for SARS-CoV-2 variants to establish infection has not been investigated.

Previous studies have demonstrated that SARS-CoV-2 is stable at room temperature on a range of surfaces for up to 96 h, on porcine skin for up to 24 h, and human skin for 9 h [40,41]. Additional studies have also investigated the effect of surface temperature and virus stability with the findings suggestive of an inverse correlation [41,42]. However, limited studies have investigated the effect of different SARS-CoV-2 variants on skin and skin-to-skin virus transference. Porcine skin is similar in morphology, hair density and sebum content to that of humans, and is therefore accepted as a model for human skin [41]. As such, virus survival on skin and skin-to-skin transference of different SARS-CoV-2 variants (Alpha, Beta, Gamma, Delta) were investigated using porcine skin in this study. In addition, ferrets were experimentally exposed via oro-nasal contact to porcine skin contaminated with the Delta variant (B.1.617.2) in a ‘wet’ format representing infectious virus droplets in suspension from a recent cough or sneeze, or ‘dry’ format representing virus particles deposited on the surface for a longer duration and are no longer in suspension or visible by eye. The infectivity of SARS-CoV-2 Delta variant following two different routes of exposure via contaminated skin was compared to a direct intranasal infection. Virus contamination in the environment, including the generation of infectious bioaerosols was also assessed. This study provides information on SARS-CoV-2 transmission in scenarios where infection mediated by skin to oro-nasal contact such as allogrooming among infected mustelid species, or when infected persons cough/sneeze onto their bare elbow/forearm skin, in line with WHO, CDC, and UK government advice [43], and then touch other parts of their skin, or mucous membranes of companion or other animals. These data contribute to the risk assessment of skin as a fomite in the context of SARS-CoV-2 virus variants survival, transference, and transmission.

## Methods

### Viruses and cell lines

The SARS-CoV-2 strains used were representative of four different variants: SARS-CoV-2/England/205080329/2020 Pango Lineage B.1.1.7 ‘Alpha variant’; SARS-CoV-2/England/205280030/2020 Pango Lineage B.1.351 ‘Beta variant’; SARS-CoV-2/England/520336_B1_P0/2021 Pango Lineage P.1 ‘Gamma variant’; SARS-CoV-2/England/21178070901/2021 Pango Lineage B.1.617.2 ‘Delta variant’. All viruses were kindly gifted by Professor Wendy Barclay at Imperial College London. The Pango lineages were defined according to the phylogenetic assignment of named global outbreak lineages (PANGOLN; https://cov-lineages.org/resources/pangolin.html). For cell culture, virus growth, and virus titration, Vero hSLAM cells (APHA) and Dulbecco’s modified Eagle’s media (DMEM, Gibco) containing 2% (v/v) foetal calf serum (FCS), 100 units ml^−1^ penicillin and 1000 µg ml^−1^ streptomycin (all Gibco) were used (hereafter referred as virus growth media). Dulbecco’s phosphate buffered saline (DPBS, Gibco) was used where stated.

### Collection, assessment, and preparation of porcine skin model

Porcine skin from three anatomical sites (axilla, hip and inguinal region) were sampled opportunistically from 3 month-old male Large White cross Landrace pigs that had been culled at the end of a vaccine potency trial. The skin was assessed macroscopically at necropsy and histologically by a veterinary pathologist.

### Evaluation of virus survival on skin

To avoid altering the biochemical composition (including lipids and proteins) on the skin surface, the porcine skin was not treated with disinfectants or antiseptic agents prior to the experiments. Following sampling at post-mortem, porcine axillary skin was processed within 1–3 days which involved cutting into approximately 0.25 cm^2^ squares and placing into 2 ml o-ring tubes. Each virus variant, standardised to a viral titre of 2.4×10^3^ TCID_50_, was applied as a 50 µl viral suspension to the epidermal side of the porcine skin sections and incubated at 35.2 °C, mimicking human skin temperature [44]. A ‘no skin’ control was used in parallel where 50 µl of the same viral suspension was placed into empty 2 ml o-ring tubes. At 0 h, 6 h, 24 h, 48 h, and 72 h post-incubation, virus growth medium was added to three replicate samples (with and without porcine skin) and then frozen at −80 °C. At the end of the study, total RNA was extracted and tested for SARS-CoV-2 by RT-qPCR as described below, and media from the ‘no skin’ controls were titrated by TCID_50_.

### Evaluation of skin-to-skin virus transference

Porcine skin was cut into approximately 1 cm^2^ squares and placed onto a sterile petri dish. Alpha, Beta, Gamma and Delta SARS-CoV-2 variants (1–6×10^4^ TCID_50_ ml^−1^) were added, in triplicate, to the epidermal aspect of the porcine skin as either a 1 µl droplet or as a bioaerosol generated from 100 µl viral suspension using a mucosal atomisation device (MAD, Teleflex) as described [39]. The skin sections were handled with sterile tweezers and used to sequentially contact ten other 1 cm^2^ sections of porcine skin, in immediate succession, on the epidermis before placing into a bijou containing 1 ml of virus growth media. To quantify the virus, the media from each sample was tested for SARS-CoV-2 RNA by RT-qPCR, as described below, titrated by TCID_50_, as described below, and compared to the original inoculum viral titre.

### Animal procedures and sampling

General anaesthesia of ferrets was performed using 4.5% isoflurane (Zoetis, Leatherhead, UK) prior to virus inoculation, virus exposure or at sample collection. Blood sample collections were performed under intravenous anaesthetic and analgesic with a single subcutaneous injection of medetomidine (0.04 mg kg^−1^, Vetoquinol, Towcester, UK) and butorphanol (0.1 mg kg^−1^, MSD Animal Health, Milton Keynes, UK). This was followed by reversal of medetomidine sedation using a subcutaneous injection of atipamezole hydrochloride (0.4 mg kg^−1^, Vetoquinol). Prior to the virus inoculation or exposure studies, a nasal wash sample and blood sample was collected from each ferret and tested to confirm absence of SARS-CoV-2 viral RNA (active infection) or specific SARS-CoV-2 antibodies (previous exposure). During the study, temperature (measured using a subcutaneous Biothermal Identichip, Destron Fearing, Dallas, TX, USA), weight, and clinical signs were monitored twice daily. Clinical samples were collected every other day from 2 days post-infection or exposure (dpi/dpe), until shedding ceased (up to 24 dpi/dpe). Clinical samples included nasal washes in DPBS, oropharyngeal (throat) swabs (MWE, Corsham, UK), rectal swabs, and fur swabs as previously described [38] and SARS-CoV-2 viral RNA (vRNA) was quantified by RT-qPCR. At the end of the study, end-point clinical samples were acquired and whole blood was collected into vacutainers, through a terminal cardiac puncture. Whole blood was allowed to clot in the vacutainers and then centrifuged at 4800 ***g*** for 6 min. The serum was aspirated and aliquoted into new tubes, heat treated at 56 °C for 30 min, and stored at −20 °C prior to serological testing. Post-mortem examination was undertaken on one representative ferret from each experiment. Respiratory turbinates, cervical trachea, thoracic trachea, left lung (cranial and caudal pool) and right lung (cranial and caudal pool) tissues were collected and a portion processed for RT-qPCR with the remaining fixed in 10% neutral buffered formalin, processed by routine histology method [45] and stained with haematoxylin and eosin stain for histopathology evaluation.

### Exposure to SARS-CoV-2 on skin using the ferret model

Ten male ferrets (*Mustela furo*) (approximately 6–12 weeks of age) were randomly assigned and housed in two groups of five, Group A and B (Fig. S1a, available in the online version of this article). Group A were exposed to ‘wet virus’ on skin: dropwise addition of 1 ml SARS-CoV-2 Delta variant (1.5×10^5^ TCID_50_) on porcine skin and immediately applied by rubbing on the oro-nasal surfaces of the ferrets for 30 s. Group B were exposed to ‘dry virus’ on skin: the same amount of SARS-CoV-2 Delta variant virus and allowed to dry on porcine skin for at least 30 min at room temperature inside a microbiological safety cabinet (MSC), before being applied in a similar way to the oro-nasal surfaces of the ferrets for 30 s. Visual inspection of the porcine skin was used to confirm that all the viral inocula had dried before exposure of the ferrets. The five ferrets from each group were then housed together and sampled every 2 days as described until viral clearance, determined by a lack of vRNA detection.

### Intranasal inoculation of ferrets and direct contact transmission

Twelve male ferrets (*Mustela furo*), approximately 6–12 weeks of age, were housed in four groups of three animals, with each group considered as a single replicate. Each group were separated from each other by metal cage surfaces or solid Perspex dividers (Fig. S1b). Within each group, one ferret was directly inoculated by dropwise intranasal instillation of 1.5×10^5^ TCID_50_ SARS-CoV-2 Delta variant. The directly inoculated ferrets were then co-housed with two naïve contact ferrets to assess viral transmission. All ferrets were sampled every 2 days and viral shedding assessed as described for SARS-CoV-2 vRNA quantification.

### Air sampling

A button air sampler (SKC Ltd., Dorset, UK) containing a 25 mm water-soluble gelatin filter (SKC Ltd.) with a 3 µm pore size connected to an Apex2 standard air sampling pump (Casella Solutions, Bedford, UK) was affixed to the inside ceiling of four cages housing the ferrets directly infected intranasally with SARS-CoV-2 Delta variant (Fig. S1b; Groups A, B, C and D). The sampling pumps were run with a flow rate of 2 litres minute^−1^ for up to 7 h to collect samples of the air within each cage. Gelatin filters from the cage air samplers were added to 1 ml media and dissolved prior to extraction. In addition to air sampling within each cage, a Coriolis Micro air sampler (Bertin Instruments, Acoem, Tewkesbury, UK) was attached to a tripod and placed in the animal room to collect samples of air within the room as an entire air space (approximately 46 m^3^) with an air flow rate of 150 litres minute^−1^ for up to 7 h into DPBS. The four cages that housed the ferrets had open bar cage fronts and perforated dividers on the non-adjacent sides to allow bioaerosols to enter the room air space. The animal room was ventilated under negative pressure with up to 20 changes per hour of conditioned air. The room temperature fluctuated between 19–22 °C with 40–60% relative humidity recorded over the course of the experiment. Liquid samples from the room air sampler were added directly to lysis buffer for extraction and vRNA testing as described.

### Extraction of RNA and detection of SARS-CoV-2 by RT-qPCR

Samples were processed, extracted, and tested as described previously [38] using the MagMAX Total Nucleic Acid Isolation kit (Thermofisher Scientific, Loughborough, UK) and the Kingfisher Flex System (Thermofisher Scientific) according to manufacturer’s instructions. A SARS-CoV-2 E-gene real time RT-qPCR was used to detect SARS-CoV-2 vRNA [46] and was quantified using a ten-fold dilution series of *in vitro* transcribed RNA for Wuhan coronavirus (2019-nCoV) targeting the E gene region (EVAg; https://www.european-virus-archive.com/nucleic-acid/wuhan-coronavirus-2019-e-gene-control) and expressed as copies per microlitre. The limit of detection of the test is was based on the equivalent to a cut-off of 37 quantification cycles (Cq).

### Virus titration via TCID_50_

Virus stocks and selected clinical samples were titrated as described [39] using 80–90% confluent Vero hSLAM cells. Virus titre was calculated using the Spearman-Karber method, reported as tissue culture infective dose 50% (TCID_50_).

### Serum processing and serological testing

Blood was collected from each animal, stored overnight at 4 °C to encourage clotting, and centrifuged at 800 ***g*** for 5 min. The serum fraction was removed and heated at 56 °C for 30 min then stored at 4 °C until required or archived at −80 °C. Serum samples were tested using the ID Screen SARS-CoV-2 Double Antigen Multi-species ELISA (IDVet) according to manufacturer’s instructions and a virus neutralisation test (VNT) as described previously [47] using 100 TCID_50_ of the inoculum SARS-CoV-2 (SARS-CoV-2/England/21178070901/2021 Pango Lineage B.1.617.2 ‘Delta variant’). Positive control serum was used from pigs inoculated with a ‘pre-variant’ B1 lineage SARS-CoV-2.

### Virus sequencing and analysis

For whole genome sequencing (WGS), vRNA was converted to double-stranded cDNA using the NEBNext ARTIC SARS-CoV-2 RT-PCR Module (New England Biolabs, Ipswich, MA, USA). Library preparation was performed using the Nextera DNA Library Prep kit (Illumina, Cambridge, MA, USA) and sequenced using the NextSeq System (Illumina) according to manufacturer’s instructions. Paired-end Illumina reads were assembled using a custom reference guided alignment script (https://github.com/APHA-VGBR/WGS_Pipelines/blob/7f73c31629f483994b8aa366e157028abf69f824/RefGuidedAlignment_Public.sh) using an early Delta genome sequence (GISAID accession no. EPI_ISL_5283939). Sequence outputs were aligned using MAFFT version 7.427 [48], visualized using mega-X [49], and sequence variants determined using flutile (https://github.com/flu-crew/flutile). The sequences were compared to the inoculum sequence (SARS-CoV-2/England/21178070901/2021 Pango Lineage B.1.617.2 ‘Delta variant’) for mutational analysis.

### Statistical analysis

All statistical analyses were performed using Graphpad Prism v8. A two-way ANOVA was used to compare viral variants survival in the presence or absence of porcine armpit skin. Dissociation one phase exponential decay was used to calculate the best-fit half-life values. Area under curve (AUC) analysis was compared using the Mann-Whitney U test with *P*<0.05 considered statistically significant.

## Results

### *In vitro* studies

#### Establishing an appropriate anatomical region of the porcine skin

**Fig. 1.**
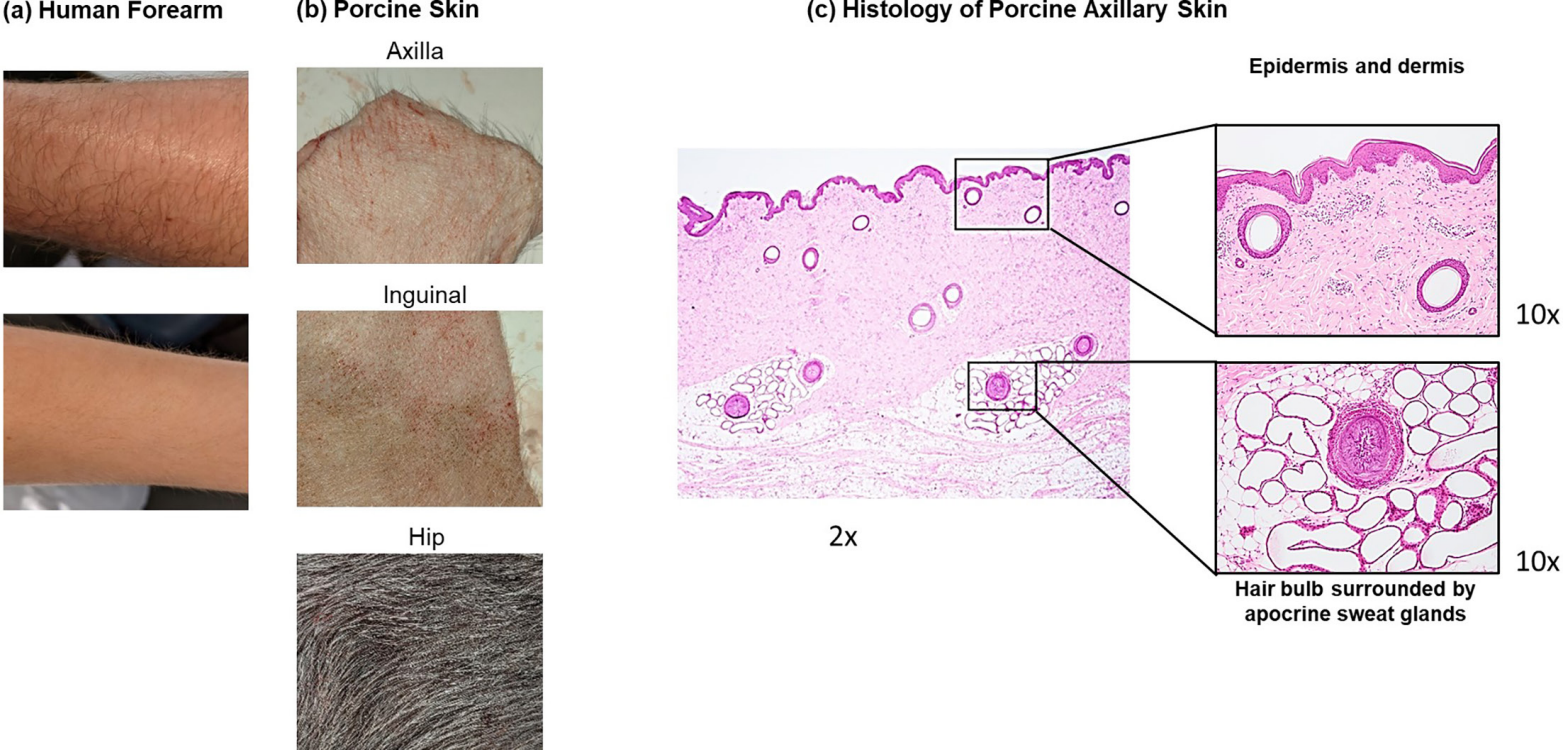
Comparison of the human forearm (**a**) with three anatomical sites of porcine skin (b; axillary, inguinal, and hip) visually showing similarities between human forearm and porcine axillary skin. Histological examination of the haematoxylin and eosin staining of porcine axillary skin (**c**) revealing similar composition of epidermal and dermal skin layers to human skin.

To use porcine skin as a model for human skin to assess SARS-CoV-2 variants survival, we sought to identify an anatomical site from which porcine skin was similar in structure and composition to human forearm skin. The skin of human forearm (Fig. 1a) was covered with sparse to moderately dense hairs, a feature similarly observed in the axillary and inguinal skin of the pig (Fig. 1b). In contrast, the dorsum of pigs, including the shoulder and hip skin, was covered with very coarse hairs and was therefore ruled out. Histological examination of porcine axillary skin (Fig. 1c) demonstrated that the epidermis was 4–5 cells thick, comprising of a thin stratum corneum, and stratum granulosum, spinosum and basale, similar to human skin [50]. Apocrine sweat glands were present alongside hair follicles within the deep dermis, while sebaceous glands and eccrine sweat glands, typical components of the human dermis such as those found in the forearm, were absent. Based on visual and histological examinations, as well as considering the ease of dissection and tissue harvesting, particularly with the lesser amount of underlying subcutaneous fat in the axillary region compared to the inguinal region, the axillary skin was chosen for investigating the survival and transference of SARS-CoV-2.

#### Virus survival on skin

**Fig. 2.**
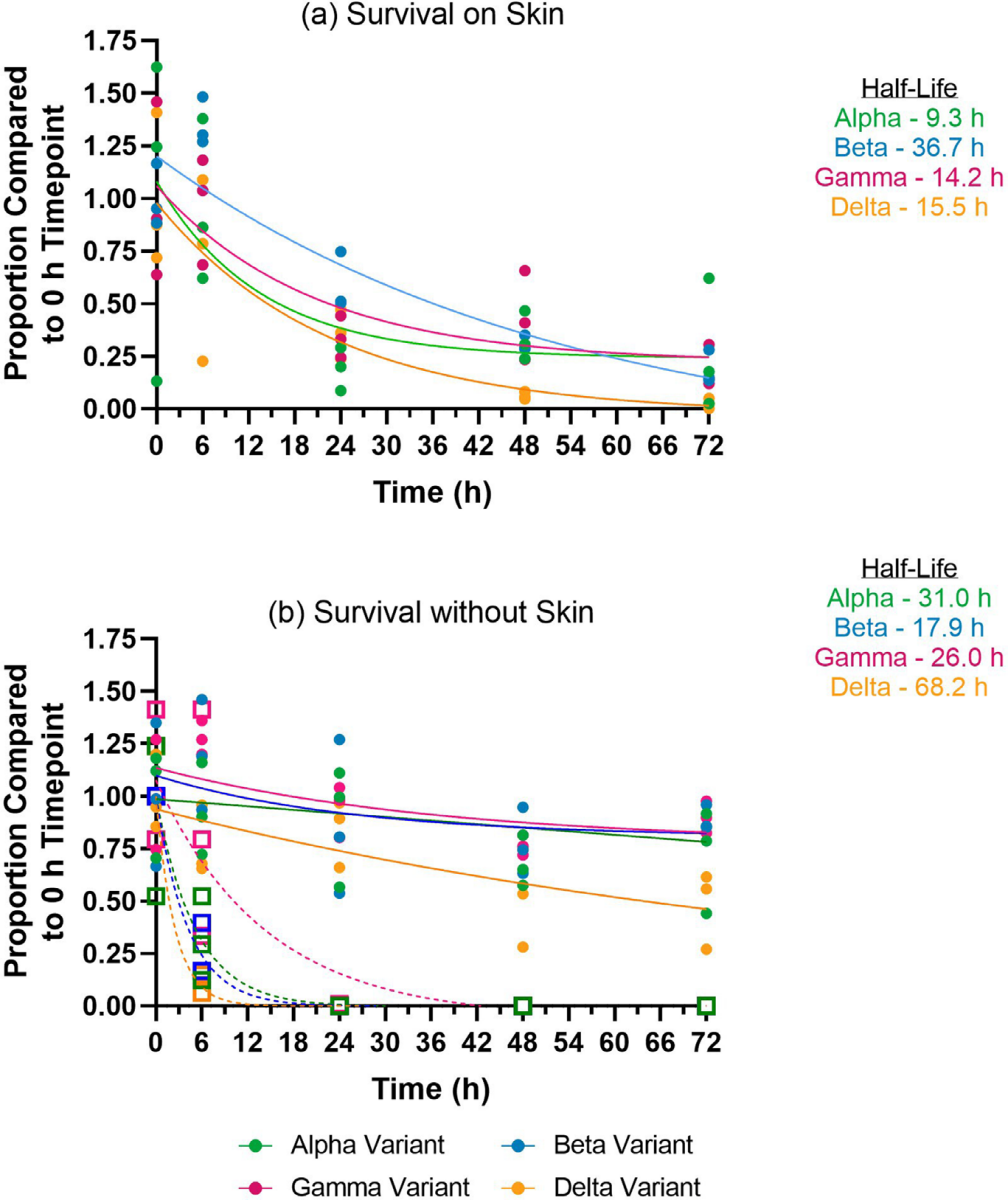
Survival of SARS-CoV-2 variants on porcine skin (**a**) and without porcine skin (**b**). Detection by SARS-CoV-2 E-gene RT-qPCR (circles; solid lines) and TCID_50_ ml^−1^ (squares; dotted lines) represented as a proportion compared to 0 hour (h) timepoint. The half-life is determined based on the RT-qPCR results by dissociation one phase exponential decay.

To assess virus survival on the skin, SARS-CoV-2 vRNA and live virus were measured over a 72 h period at skin-surface temperature. SARS-CoV-2 vRNA was detected up to 72 h after incubation at 35.2 °C, both in the presence or absence of porcine skin (Fig. 2). However, the proportion of detectable vRNA compared to the 0 h timepoint was less on skin than the ‘no skin’ controls. On porcine skin, the average proportion of vRNA at the 72 h timepoint was 27% for Alpha variant, 19% for Beta variant, 19% for Gamma variant, and 3% for Delta variant. In the absence of skin, the average proportion of vRNA at the 72 h timepoint was 72% for Alpha variant, 89% for Beta variant, 90% for Gamma variant, and 48% for Delta variant, higher than in the presence of skin. Beta and Gamma variants on skin demonstrated a significant reduction in proportion of vRNA at the 72 h time-point compared to the ‘no skin’ controls (*P*=0.003 and *P*=0.007, respectively). The half-life for vRNA detection in the presence of porcine skin was 9.3 h for Alpha variant, 36.7 h for Beta variant, 14.2 h for Gamma variant, and 15.5 h for Delta variant. Whereas the half-life for vRNA detection in the absence of porcine skin was 31.0 h for Alpha variant, 17.9 h for Beta variant, 26.0 h for Gamma variant, and 68.2 h for Delta variant. Assessment of infectious viral titre over time was also attempted. The infectious viral titre of the samples collected in the absence of porcine skin was determined and infectious virus was detected up to 24 h incubation at 35.2 °C (Fig. 2b). Detection of infectious virus differed between variants where Alpha, Beta, and Delta variants were detected up to 6 h, whereas a low average proportion of Gamma variant was detected up to 24 h (0.05%). Infectious virus was not recoverable in the presence of skin.

#### Skin-to-skin transference of virus

**Fig. 3.**
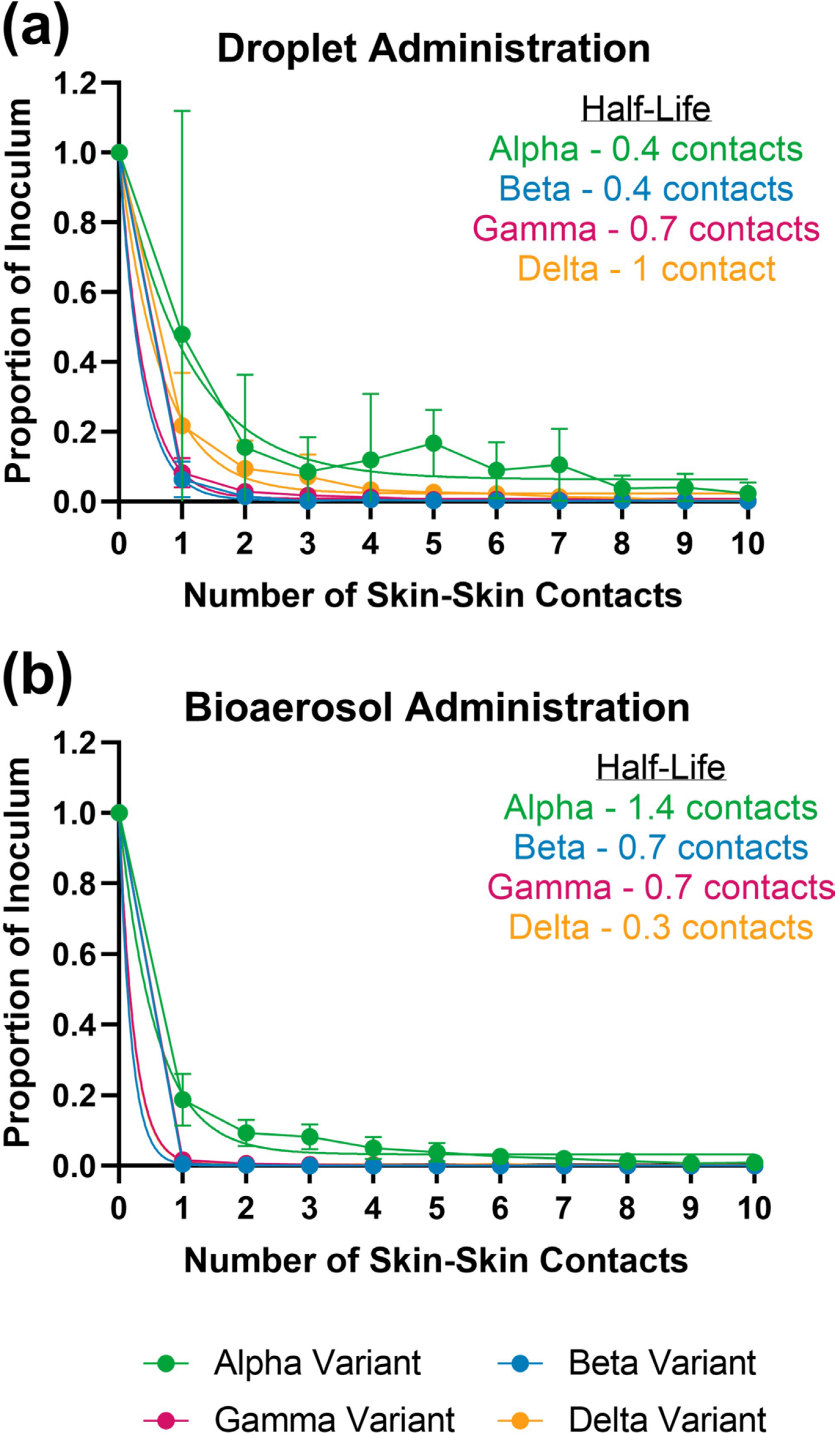
Detection of SARS-CoV-2 variants vRNA after ten skin-to-skin touch transfers following (**a**) droplet administration of 1 µl virus or (**b**) bioaerosol administration using 100 µl viral suspension in a mucosal atomisaion device. Detection of vRNA by E-gene RT-qPCR and expressed as proportion relative to inoculum.

To assess the presence of virus after a number of skin-to-skin transfers, SARS-CoV-2 variants were first administered to the epidermal side of the porcine skin as either a 1 µl droplet or as a bioaerosol generated from 100 µl viral suspension using a MAD. These skin sections were then used to sequentially touch ten other 1 cm^2^ sections of porcine skin on the epidermal-to-epidermal side and recovery of total RNA from these sections were attempted. SARS-CoV-2 RNA from all variants were detectable on skin following multiple skin-to-skin contacts. vRNA was detectable at low levels (Cq value <37.0) by RT-qPCR after ten skin-to-skin transfers following both virus administration methods (Fig. 3). After one skin-to-skin transfer, when compared to the amount of virus added to the initial piece of skin, recovery of different variants ranged from 6.38–47.98% for droplet administration (Fig. 3a) and 0.57–18.77% for bioaerosol administration (Fig. 3b). After ten skin-to-skin transfers, when compared to the amount of virus added to the initial piece of skin, recovery of different variants ranged from 0.14–2.35% for droplet administration (Fig. 3a) and 0.01–0.99% for bioaerosol administration (Fig. 3b). For both administration methods, recovery of the Alpha variant was highest. Assessments of the half-life of the number of skin-to-skin contacts were consistent between administration methods and ranged between 0.3–1.4 skin-to-skin contacts.

### *In vivo* studies

#### Experiment 1. Exposure of ferrets to SARS-CoV-2 Delta variant contaminated porcine skin

**Fig. 4.**
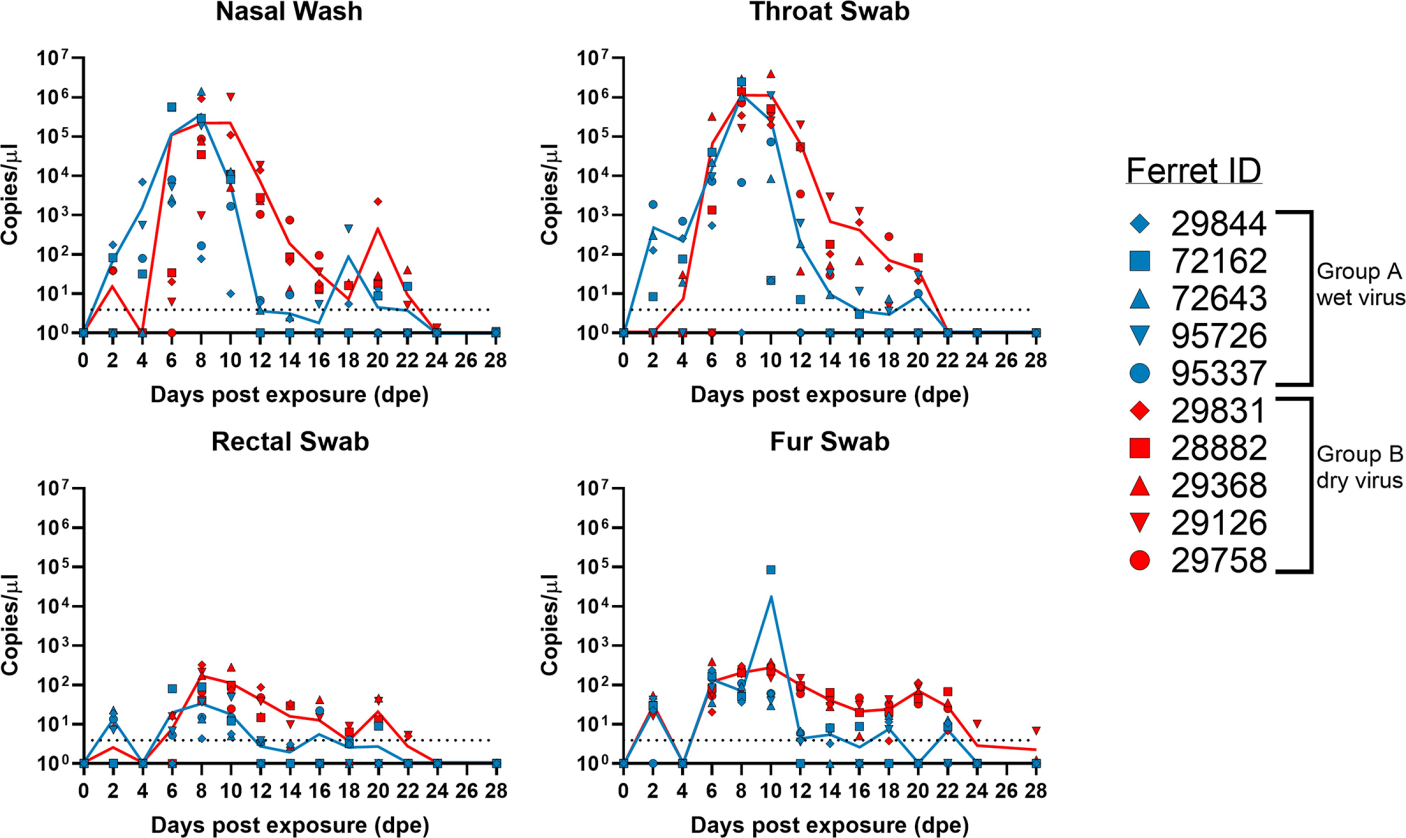
SARS-CoV-2 vRNA detection following ferret exposure to porcine skin with wet virus (Group A; blue symbols and line), or dry virus (Group B; red symbols and line). Clinical samples taken from ferret nasal washes and swabs from throat, rectum and fur. vRNA was detected using the SARS-CoV-2 E-gene RT-qPCR and quantified with a ten-fold dilution series of *in vitro* transcribed RNA expressed as copies per microlitre. Dotted horizontal lines indicate the limit of detection equivalent to a cut-off of 37 quantification cycles (Cq).

SARS-CoV-2 vRNA was detected in the nasal washes and throat swabs of ferrets exposed to ‘wet virus’ on skin (Group A; *n*=5) and ferrets exposed to ‘dry virus’ on skin (Group B; *n*=5) from two dpe (Fig. 4). Average peak shedding at these sites differed slightly between groups. In group A, peak shedding occurred between 6–8 dpe. In group B, peak shedding occurred between 6–12 dpe. In both groups, shedding ceased after 22 dpe as indicated by a drop in vRNA levels below the limit of detection. Whilst the duration of shedding was similar for both groups, Group B showed elevated SARS-CoV-2 RNA levels for a longer period in both the nasal wash and throat swab samples. However, AUC comparisons showed that these differences were not significant. In general, quantified viral titres of selected clinical samples were lower in Group A compared to Group B (Table 1). In Group A, the highest viral titre was detected in the throat swab sample of ferret #72 162 at 1.12×10^4^ TCID_50_ ml^−1^ (8 dpe) and in Group B, the highest viral titre was detected in the nasal wash sample of ferret #29 126 at 2.00×10^4^ TCID_50_ ml^−1^ (10 dpe). In both groups, low vRNA loads were detected from the rectal and fur swabs. However, detection of vRNA in Group A was more sporadic whilst in Group B, vRNA was consistently detected between 8–18 dpe in the rectal swab samples and between 6–22 dpe in the fur swab samples.

**Table 1.** SARS-CoV-2 virus isolation, quantification by TCID_50_ and amino acid substitutions in the SARS-CoV-2 genome compared to the inoculum virus from selected ferret and air samples

Experiment	Group	Ferret ID and cage no.	Sample type	DPE/DPI	vRNA load (copies µl^−1^)	Viral titre (TCID_50_ ml^−1^)	SARS-CoV-2 protein				
							Nsp1	Nsp3	S	ORF3a	M
(1)Skin transference	AWet virus on skin	29844 1A	NW	4	6.98E+03	1.12E+01	–	–	–	–	–
		72162 1A	OR	8	2.47E+06	1.12E+04	–	L1486V	G142D Y453F	–	–
		72643 1A	OR	8	2.15E+06	6.32E+02	–	L1486V	G142D Y453F	–	D160Y
		95726 1A	OR	8	1.01E+06	4.74E+02	–	L1486V	G142D Y453F	–	–
		95337 1A	OR	10	7.24E+04	negative	nt	nt	nt	nt	nt
	BDry virus on skin	29831 1B	NW	8	9.19E+05	1.12E+03	–	L1486V	G142D Y453F	–	–
		28882 1B	OR	8	1.38E+06	6.32E+03	–	L1486V	Y453F	–	–
		29368 1B	OR	10	4.00E+06	6.32E+02	Q22R	Y1323C	G142D Y453F N959D	T175I	–
		29126 1B	NW	10	1.00E+06	2.00E+04	Q22R	Y1323C	Y453F	T175I	–
		29758 1B	OR	8	7.11E+05	3.56E+03	–	L1486V	G142D Y453F	–	–
(2)Transmission study	Directly infected	29744 2A	OR	6	2.50E+05	3.56E+01	–	L1486V	Y453F	–	–
	Contact	28913 2A	OR	8	4.84E+05	7.11E+02	–	L1486V	Y453F	–	–
	Contact	72522 2A	OR	8	7.62E+05	3.56E+01	–	L1486V	G142D Y453F	–	–
	Air filter	2A	GF	6	3.30E+02	negative	–	L1486V	Y453F	–	–
	Directly infected	73008 2B	OR	8	1.09E+05	negative	–	–	G142D F486L	–	–
	Contact	29732 2B	NW	16	5.06E+06	6.32E+03	–	L1486V	G142D Y453F	–	–
	Contact	29812 2B	OR	14	7.08E+04	1.12E+01	–	L1486V	Y453F	–	–
	Air filter	2B	GF	5	8.60E+01	nt	nt	nt	nt	nt	nt
	Directly infected	72781 2C	NW	2	1.79E+05	1.12E+03	–	–	–	–	–
	Contact	72126 2C	OR	8	9.76E+05	1.12E+03	Q22R	Y1323C	G142D Y453F	T175I	–
	Contact	29571 2C	NW	6	7.88E+05	1.12E+03	Q22R	Y1323C	Y453F	T175I	–
	Air filter	2C	GF	6	4.04E+02	negative	Q22R	Y1323C	G142DY453F	T175I	–
	Directly infected	73138 2D	NW	2	1.62E+04	3.56E+02	–	–	–	–	–
	Contact	29188 2D	NW	10	2.17E+06	1.12E+04	–	L1486V	G142D Y453F	–	–
	Contact	29202 2D	NW	12	4.37E+05	3.56E+02	–	L1486V	G142D Y453F	–	–
	Air filter	2D	GF	11	2.70E+02	negative	–	L1486V	N959D	–	–

**Fig. 5.**
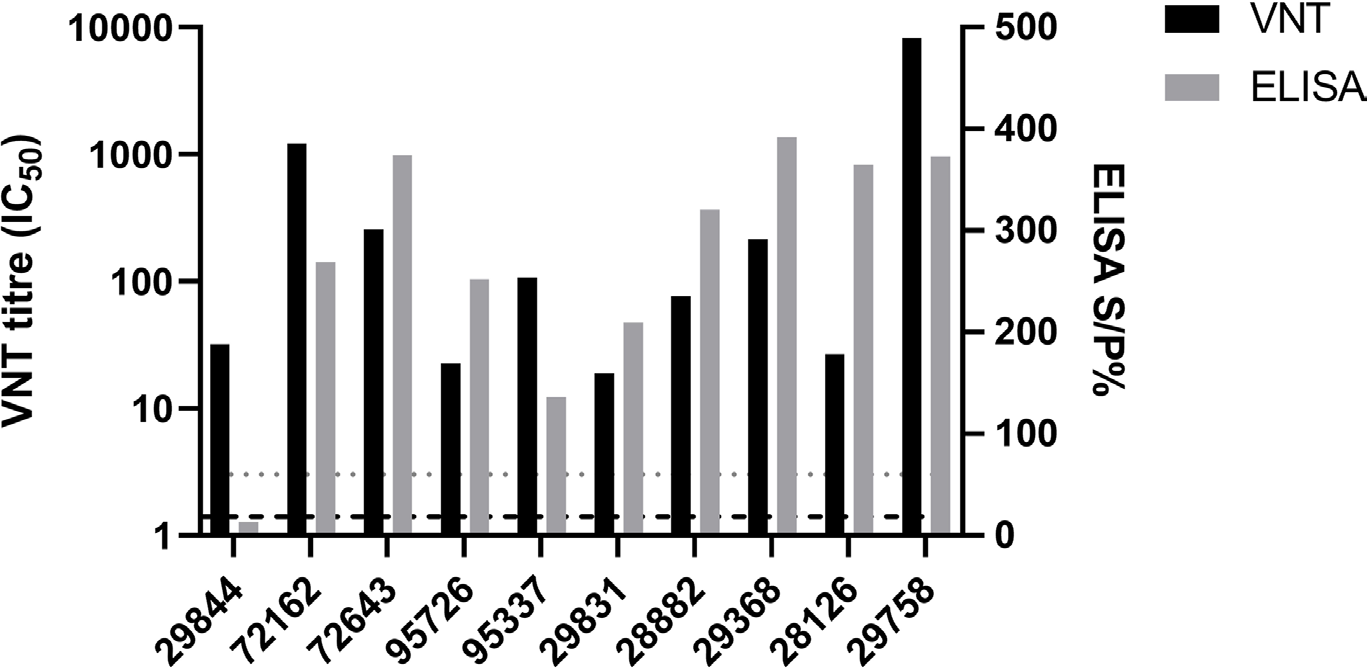
Serological analysis of ferrets exposed to or directly infected with SARS-CoV-2 Delta variant. Homologous SARS-CoV-2 virus neutralisation test (VNT) titres and ID Screen SARS-CoV-2 Double Antigen Multi-species ELISA (IDVet) from serum collected from experiment 1. Neutralisation titres are displayed as inhibition concentration 50% (IC_50_) calculated using the Spearman-Karber method. The dashed horizontal line indicates the limit of detection for neutralisation equivalent to 1.41 IC_50_. The dotted line indicates the positivity cut off ratio equivalent to 60%.

-, no amino acid changesDPE, days post-exposure, Cq, quantification cycle; DPI, days post-infection; GF, Gelatin filter; NW, Nasal wash; OR, Oral swab; nt, not tested;

Serological testing by ELISA and VNT on sera collected from each ferret at the end of the study (28 dpe) demonstrated that all ten ferrets seroconverted (Fig. 5). One ferret exposed to wet virus on porcine skin (Group A) was negative by ELISA but demonstrated SARS-CoV-2 neutralising antibodies by VNT. No alteration of body temperature or weight outside of normal fluctuations or overt clinical signs were observed in the ferrets throughout the study. No gross pathology changes were observed at post-mortem examination. Histopathological assessment revealed scattered neutrophilic infiltration within the submucosa of the respiratory mucosa of the nasal turbinate of ferret #72 162 (Group A; wet virus on skin) as well as occasional loss of cilia and attenuated respiratory epithelium of the nasal turbinate of ferret #29 831 (Group B; dry virus on skin). No vRNA was detected in any of the respiratory tissues collected from these two ferrets at the end of the study.

In summary, ferrets exposed to either wet virus or dried virus on porcine skin were robustly infected with the SARS-CoV-2 Delta variant and seroconverted by 28 dpe. Viral shedding was predominantly via oro-nasal routes and resulted in contamination of fur.

#### Intranasal infection of ferrets with the Delta SARS-CV-2 variant and transmission

**Fig. 6.**
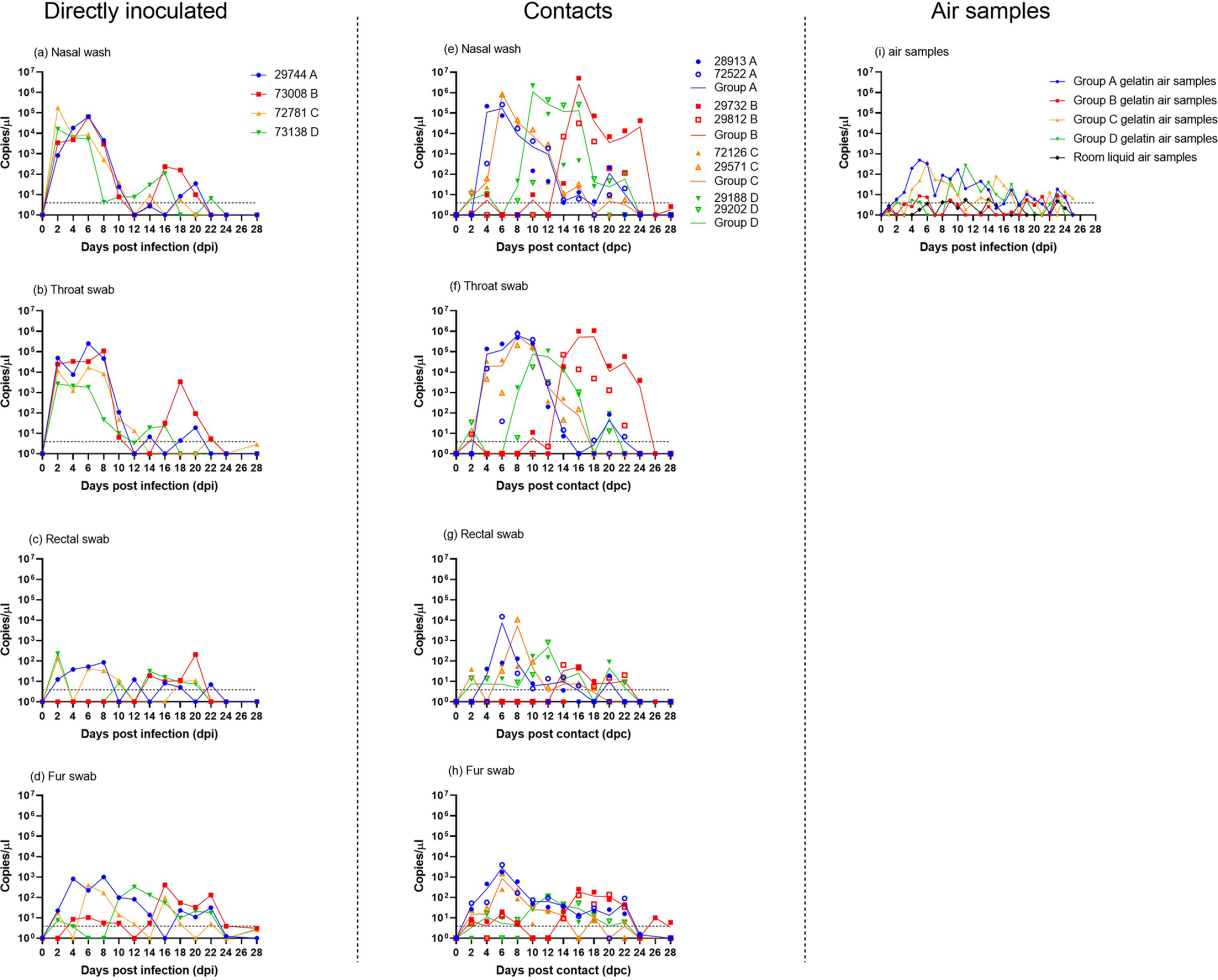
SARS-CoV-2 vRNA detection following direct intranasal infection of ferrets (**a–d**) and from ferrets placed in direct contact (**e–h**) housed in four separate groups A–D. Data plotted from each individual contact ferret (symbols) and average per group (solid line), each group represented by a different colour. Clinical samples taken from ferret nasal washes and swabs from throat, rectum and fur. SARS-CoV-2 viral RNA detection from air samples collected from within the cages of infected ferrets and from air samples collected from the room the cages were housed in (**i**). Viral RNA was detected using the SARS-CoV-2 E-gene RT-qPCR and quantified with a ten-fold dilution series of *in vitro* transcribed RNA expressed as copies per microlitre. Dotted horizontal lines indicate the limit of detection equivalent to a cut-off of 37 quantification cycles (Cq).

All 12 ferrets directly intranasally inoculated or in direct contact, demonstrated shedding from both the nasal washes and throat swabs from 2 to 22 dpi (Fig. 6). For the directly-inoculated ferrets, the peak of shedding was observed between 2–8 dpi with AUC analysis indicating no significant difference among all four ferrets (Fig. 6a, b). However, different shedding profiles were observed for the different groups of contact ferrets (Fig. 6e, f). Shedding via the oro-nasal route from contact ferrets peaked first in Groups A and C at eight dpc followed by Group D at 10 dpc and lastly Group B at 16 dpc. Interestingly, the directly-inoculated ferret in Group B #73 008 appeared to cease oro-nasal shedding between 12–14 dpi and recommence shedding between 16–22 dpi which may have contributed to the delayed shedding in the contact ferrets in Group B. Overall, the contact ferrets appeared to shed higher levels of vRNA for a longer duration compared to the directly-inoculated ferrets. Lower levels of vRNA were detected in the rectal and fur swabs (Fig. 6c, d, g, h) however a similar profile was observed with the directly-inoculated ferrets and the two contact ferrets where rectal shedding and detection of vRNA on fur was seen in Groups A and C first, followed by Group D and lastly Group B. Overall, viral titres of selected clinical samples were similar or higher in the contact ferrets compared to the directly inoculated ferret in the same group (Table 1). The highest viral titre was detected in the nasal wash of contact ferret #29 188 (Group D) at 1.12×10^4^ TCID_50_ ml^−1^ (10 dpc). This level is comparable to the highest viral titres obtained from nasal wash samples in experiment one when ferrets were exposed to virus contaminated skin.

Serological testing by ELISA and VNT on sera collected from each individual ferret at the end of the study (28 dpi) showed that all 12 ferrets had antibodies towards SARS-CoV-2 (Fig. S2). A negative ELISA result (3.73 S/P%) was obtained from ferret #73 138 (directly-inoculated ferret from Group B) however neutralising antibodies were detected and this ferret shed virus from 2 to 16 dpi providing evidence that it was infected. No alteration of body temperature or weight outside of normal fluctuations or overt clinical signs were observed in any of the ferrets during this experiment. No gross pathology changes were observed at post-mortem examination. Histopathology revealed mild lymphocytic cuffing of pulmonary vessels and occasionally scattered in the alveolar walls of the directly inoculated ferrets #29 744 (Group A) and #73 008 (Group B). Additionally, rare lymphocytic perivascular cuffing and bronchiolar-associated lymphoid tissue was noted in one of the lung lobes in contact ferret #28 913 (Group A). These findings suggested antigenic stimulation in the lung. No histopathological changes were seen in contact ferret #29 732 (Group B), although vRNA was detected at a low level in the respiratory turbinate (1.14 copies µl^−1^).

Overall, ferrets directly inoculated with SARS-CoV-2 Delta variant became robustly infected, subsequently contaminated the fur, and virus was transmitted to all contact ferrets.

### Detection of viral bioaerosols from SARS-CoV-2 infected ferrets

Viral RNA was detected in the air samples collected from the cages housing directly-inoculated and contact ferrets (Fig. 6i). The peak of vRNA detection in Groups A, C and D mirrored the detection of vRNA in these groups from the oro-nasal shedding, albeit at much lower levels. The vRNA levels detected were comparable to the fur swabs, providing further evidence for environmental contamination. Very low vRNA levels were detected from the air sampler in the cage housing Group B ferrets (Fig. 6i). Infectious virus was not obtained from the air samples from cages A, C or D (Table 1). Low levels of vRNA were detected from the liquid air sampler in the room demonstrating the potential for environmental contamination beyond the immediate area within which the infected ferret was residing, however, this was at even lower levels than the air sampled from within the cages and virus infectivity was not established.

#### Genetic evolution of the SARS-CoV-2 Delta variant following infection and transmission in ferrets and environmental contamination

To investigate potential adaptive mutations that may have arisen following replication in ferrets, WGS was performed on clinical samples from each ferret in all experiments and compared to the genome of the inoculum virus. The clinical sample with the lowest Cq value and/or the highest virus titre (Table 1) from each ferret or air sample was selected for WGS. Genome sequences from 24 samples were obtained with at least 90% genome coverage for comparison with the inoculum virus sequence. Following ferret virus exposure, direct inoculation or transmission, nine amino acid substitutions were observed. These were identified in the Nsp1 (Q22R [*n*=5/24]), Nsp3 (Y1323C [*n*=5/24]; L1486V [*n*=15/24]), S (G142D [*n*=13/24]; Y453F [*n*=19/24]; F486L [*n*=1/24]; N959D [*n*=2/24]), ORF3a (T175I [*n*=5/24]) and M (D160Y [*n*=1/24]) protein sequences (Table 1). The group administered with dry virus on skin resulted in more genetic changes compared to the group administered with the wet virus (Table 1). In experiment two, the contact ferrets had more genetic changes compared to the directly inoculated ferrets from each group (Table 1). Substitutions observed in the air samples collected from within cages A, C and D matched those found in the ferrets that were directly inoculated or infected through direct contact.

## Discussion

The rapid spread of SARS-CoV-2 among humans since the start of the COVID-19 pandemic in 2020 was attributed to virus transmission through direct exposure to infectious droplets or indirect exposure via fomites [28]. To prevent onward transmission, covering the nose and mouth with a face mask was advised to reduce the spread of particles carrying the virus. In scenarios where face masks were not worn it was also advised to cough and sneeze into the crook of the elbow instead of a hand, if a tissue was not available, to limit contamination of surrounding surfaces. Studies using early SARS-CoV-2 variants have investigated virus survival on various surfaces to understand virus persistence. Reports indicated increased SARS-CoV-2 stability on cardboard and plastic compared to SARS-CoV [31] and an eight-fold longer survival time of SARS-CoV-2 on stainless steel, borosilicate glass, and polystyrene surfaces compared to influenza A virus [40]. In addition, SARS-CoV-2 survival on skin, clothing, and bank notes was determined to be mostly temperature dependent, whereby, for all three surface materials, infectious virus was retained for up to 96 h at 4 °C, whilst no recoverable virus was detected after 4 h at 37 °C [41]. This is the first report that investigates the difference in survival and transference on skin, at human skin-surface temperature (35.2 °C), with four SARS-CoV-2 variants. First, porcine axillary skin was determined as a suitable surrogate model for human skin due to similarity in the anatomical features (Fig. 1). Porcine skin has also been used as a model for human skin in other studies [41,51] and similarities between porcine skin and human skin have been described previously [52]. For all variants, vRNA was detected up to 72 h, however, the proportion of vRNA detected was markedly reduced in the presence of skin versus the ‘no skin’ controls (Fig. 2) from 24 h onwards. In the case of Beta and Gamma variants, the proportion of vRNA detected at 72 h was significantly reduced on skin compared to the ‘no skin’ controls. The survival curves among the variants were not significantly different at the time points tested, however in this study, the detection of Delta variant vRNA demonstrated the lowest level of survival at 72 h on skin and Alpha variant vRNA demonstrated the highest. It is acknowledged that *ex vivo* manipulation of the porcine skin for several days may not accurately represent natural conditions and may have contributed to the accelerated decay of SARS-CoV-2 compared to survival without skin. For all variants without skin, infectious virus was not detected after the 6 h timepoint. The porcine skin was not treated with antiseptic agents prior to the experiments to minimise disruption to its physiological biochemical composition. However, this subsequently led to cell culture contamination with bacterial and fungal overgrowth, rendering infectious live virus titration unachievable. Microfiltration was attempted, but the filtrate volumes was insufficient for repeat titration. A previous study determined that SARS-CoV-2 survival was significantly reduced on human skin surfaces at 25 °C compared to other inorganic surfaces, suggesting that human skin is less of a risk for SARS-CoV-2 survival than other surfaces [40], however, we have clearly demonstrated that it is a robust transmission competent fomite. As well as the effect of virus survival on different surface matrices, survival of SARS-CoV-2 has been shown to be largely temperature dependent [41]. One study showed that infectious SARS-CoV-2 Wuhan strain was detected up to ≈9 h at 25 °C on human skin [40] with longer survival times reported with Alpha, Beta, Delta and Omicron variants up to 21 h [53], whilst another study showed that infectious SARS-CoV-2 was detected up to 4 h at 37 °C on porcine skin [41]. Based on these results, we hypothesise that at human skin surface temperature (35.2 °C), survival of each of the different SARS-CoV-2 variants tested in this study on skin would not exceed survival detected without skin and corroborate with more conservative estimates of virus viability.

Transference of virus on skin following droplet and bioaerosol contamination with four different SARS-CoV-2 variants were also assessed. The results showed that skin could act as a fomite for onward virus transmission with a half-life of 0.3–1.4 skin-to-skin contacts. This supports findings that survival on skin is short-lived compared to other non-biological surfaces [40,54] and is comparable to other studies where transference of SARS-CoV-2 to skin from contaminated surfaces was reported [51,55]. In scenarios where large droplets or bio-aerosolised virus particles are deposited on skin, such as a human forearm from a cough or sneeze, there is the potential for virus transfer via skin-to-skin touch, however, the findings show that multiple skin-to-skin contacts cannot sustain high levels of virus transfer and therefore may not contribute to a high-risk or high-frequency route of transmission.

Following on from the *in vitro* assessments of SARS-CoV-2 survival and transference on porcine skin, we sought to ascertain whether contaminated skin could act as a source of initiating SARS-CoV-2 infection in ferrets. The Delta variant was selected due to it being the most contemporaneous variant of concern at the time of the study and was shown to have increased infectivity and transmissibility properties compared to earlier variants [56]. All ferrets exposed to either wet or dried virus on porcine skin became infected as evidenced by shedding (Fig. 4) and seroconversion (Fig. 5). Infectious virus could be recovered from four out of the five (80%) ferrets exposed to ‘wet’ virus on skin and all five ferrets (100%) exposed to ‘dried’ virus on skin (Table 1). These findings suggest that residual infectious virus remained on the skin surface after 30 min despite no visible droplets observed by eye. It is acknowledged that the contaminated porcine skin-to-ferret oro-nasal skin contact performed in this experiment involved rubbing the surfaces together for 30 s which may have enhanced virus transfer [57]. However, a previous study demonstrated that transference of SARS-CoV-2 was possible, albeit at low levels, following light touch of artificial skin to a solid surface contaminated with SARS-CoV-2, even after the droplet had evaporated [55]. In our study, similar levels of vRNA were obtained from ferrets exposed to contaminated skin (Fig. 4) compared to ferrets directly intranasally inoculated with the same virus (Fig. 6a–d), demonstrating a reproducible infection. The shedding levels were also comparable to ferrets directly intranasally inoculated with earlier SARS-CoV-2 variants [38,39]. While limited to the Delta variant in this study, a contact exposure challenge method via the skin to oral-nasal route could represent an improvement of experimental studies for SARS-CoV-2 and potentially other respiratory pathogens to better mimic a natural, biologically-relevant, yet controlled, route of experimental exposure.

To further assess virus transmission through environmental contamination by allogrooming or from infected aerosols, ferrets were directly-inoculated and then housed with contact ferrets. Following direct intranasal inoculation with the SARS-CoV-2 Delta variant, all eight contact ferrets became infected as evidenced by vRNA shedding (Fig. 6) and seroconversion (Fig. S2). Among companion animals, studies have demonstrated that SARS-CoV-2 can transmit via droplets, aerosols and direct contact in ferrets, cats, and hamsters [14,33,36]. Detection of SARS-CoV-2 vRNA on the fur of the ferrets following direct inoculation in our study and previous studies [38,39] indicates environmental contamination, and that allogrooming and human-handling could facilitate onward transmission. Infectious SARS-CoV-2 particles in respiratory droplets and bioaerosols remain another source of infection and transmission among humans and animals. In our study, lower vRNA levels detected in the air samples compared to clinical samples from the ferrets (Fig. 6) is similar to a study of SARS-CoV-2 infected patients where 1.1–4.8 copies m^−3^ was detected in air samples while high viral loads (10^5^–10^8^ copies ml^−1^) were detected from cough samples [58]. Even lower vRNA levels were detected from the room air sampler, and although this demonstrated contamination beyond the immediate area where infected ferrets were residing, the limited detection could be due to the requirement of 20 air changes per hour in the animal facility and may not accurately reflect natural environmental conditions. As demonstrated in this study, vRNA load does not directly correlate with infectivity titres (Table 1) and while virus isolation from air samples were not successful in this study, reports of culturable virus could be recovered from air samples ranging from 16 to 101 gene copies m^−3^ of air [32,59]. The inability to culture virus from air samples in this study could be attributed to the collection process which may have contributed to the loss of viability or inactivation of SARS-CoV-2, as previously suggested [30,60]. Other studies have demonstrated that infectious SARS-CoV-2 could be recovered from air samples after 3 h [31] in an experimental setting although ferret exposure to respiratory droplets was seen to be inefficient [39].

In addition to human infections, SARS-CoV-2 has been detected in a range of animal species including farmed mink, farmed white-tailed deer, zoo animals, and companion animals [18]. The potential to establish a reservoir host following sustained circulation of SARS-CoV-2 infections in animal populations and the accumulation of mutations that render the virus distinct from circulating strains are a public health concern [61]. Following SARS-CoV-2 transmission events among mink in Europe, several mutations were described to be attributed to virus adaptation in a new host [25,62]. These included mustelid-adapted SARS-CoV-2 substitutions in the spike protein Y453F, F486L, and N501T. Of note, the spike protein substitution Y453F was identified in 19 samples sequenced in this study from ferrets exposed to dry or wet contaminated porcine skin, direct intranasal ferret inoculation, contact ferrets and from air samples (Table 1) as well as being observed in our previous study [38]. Another substitution identified in the spike protein, F486L, was only seen in one directly infected ferret however this has been observed in directly inoculated or contact ferrets in a previous study [39]. This mutation has also been associated with viral adaptation in mink in Denmark and the Netherlands [25,62] and resistance to neutralisation by mAbs, polyclonal sera, and convalescent plasma [63]. Among the substitutions in the spike protein, G142D was seen in 13 samples sequenced and was represented in all ferret groups including the air samples. This residue is in the N-terminal domain of the spike protein and has been described as a lineage defining mutation for the Delta variant, however it has also been determined to be an artefact of sequencing caused by a deletion in the binding site for the 72_RIGHT primer of the ARTIC schema [64]. The T175I substitution in the ORF3a protein was seen in five samples in this study from ferrets exposed to dried virus on porcine skin and from contact ferrets and the air sample from one cage. The T175I substitution has been observed previously in early lineages of SARS-CoV-2 collected in 2020 [65] and was not seen to introduce structural modifications *in silico* [66]. The substitutions seen in Nsp1 (Q22R) and Nsp3 (Y1323C; L1486V) have not been reported to alter protein function however they have been observed in Delta variant sequences from human samples available in the EpiCoV database (GISAID). The additional substitutions observed in the spike protein N959D and membrane protein D160Y remain uncharacterised but may be unique to this Delta variant and associated with adaptation to ferrets. In this study we observed that more genetic polymorphisms were detected from the ferrets exposed to dry virus on skin compared to the group with the wet virus (Table 1). Longer virus incubation periods were seen for the ‘dry’ group compared to the ‘wet’ group, which we hypothesise to be a result of lower viral load upon infection and the subsequent cause for increased mutations. Interestingly, in experiment two, the contact ferrets exhibited more mutations compared to the directly inoculated ferrets from each group which may have arisen from sustained replication. Virus genome substitutions observed in the air samples matched those found in the ferrets providing evidence for the utility of air sampling as a non-invasive method to detect virus shedding and to understand potential genetic variants that arise following infection in a particular host.

Overall, the study demonstrates that SARS-CoV-2 variants can persist on skin with capability for onward transference via skin-to-skin contact. Subsequent initiation of SARS-CoV-2 infection through a skin to oro-nasal contact route highlights the role of contaminated skin in virus infection in a population and informs the continued assessment of intervention strategies to reduce the risk of SARS-CoV-2 transmission.

## supplementary material

10.1099/jgv.0.002022 Uncited Supplementary Material 1.

## Abbreviations

APHA: Animal and Plant Health Agency
AUC: area under curve
COVID-19: coronavirus disease 2019
Cq: quantification cycle
DMEM: Dulbecco's modified Eagle's media
DPBS: Dulbecco's phosphate buffered saline
dpc: days post contact
dpe: days post exposure
dpi: days post infection
FCS: foetal calf serum
MSC: Microbiological Safety Cabinet
RT-qPCR: reverse transcriptase quantitative PCR
SARS-CoV-2: severe acute respiratory coronavirus-2
TCID50: tissue culture infective dose 50%
VNT: virus neutralisation test
vRNA: viral RNA
WGS: whole genome sequencing
WOAH: World Organisation for Animal Health
