# Supplementary material for: SARS-CoV-2 infection and transmission via the skin to oro-nasal route with the production of bioaerosols in the ferret model
Source: J Gen Virol. 2024 Sep 18;105(9):002022. doi: 10.1099/jgv.0.002022 (PMC11410047; doi:10.1099/jgv.0.002022)
Supplement: Uncited Supplementary Material 1. [file jgv-105-02022-s001.pdf]

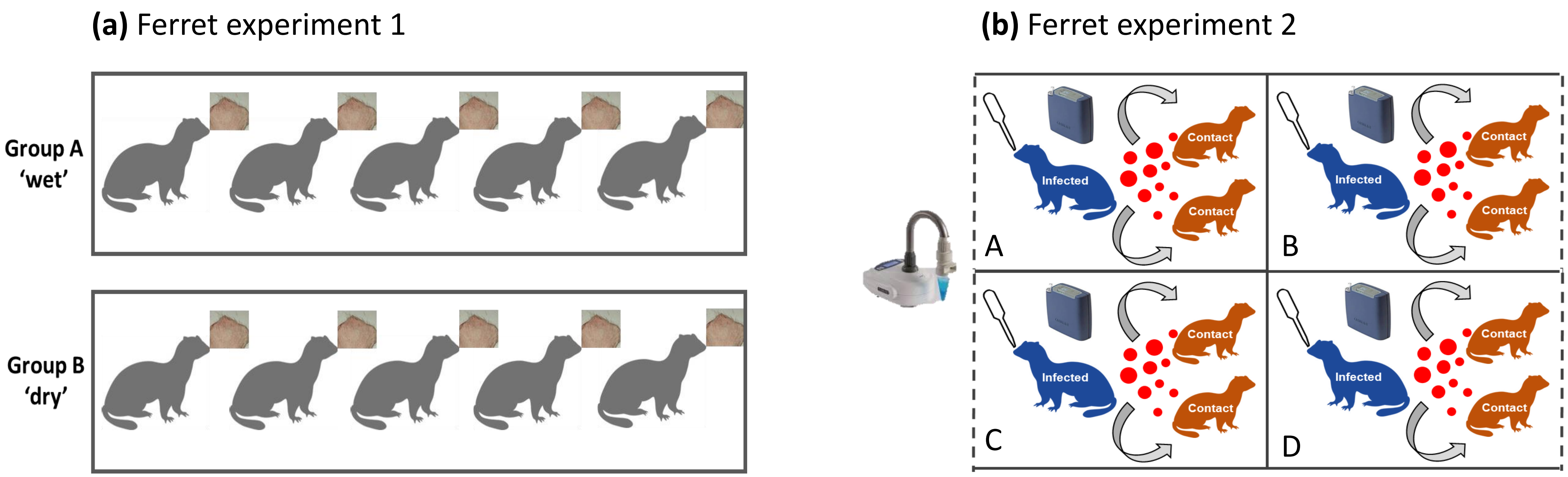


Figure S1. Ferret experimental design. In experiment 1 (a) five ferrets were housed together and individually exposed to 'wet' virus on porcine skin (Group A). A separate group of five ferrets were housed together and exposed to 'dry' virus on porcine skin (Group B). In experiment 2 (b) four ferrets were directly inoculated intranasally with virus and housed separately in four different cages (Group A, B, C and D) each with two naive contact ferrets. An air sampler was placed in each cage and one placed in the room outside the cages.

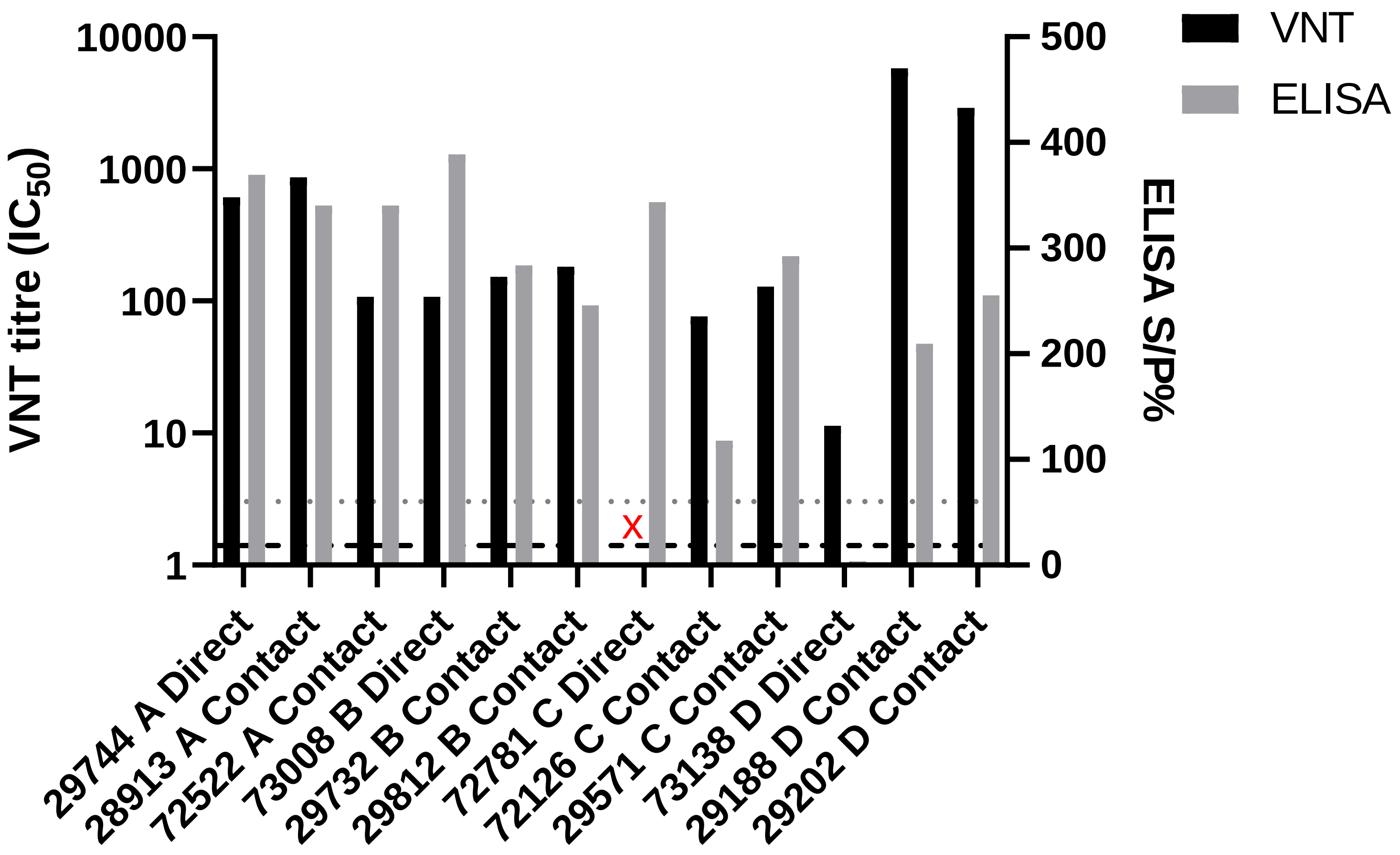


Figure S2. Serological analysis of ferrets directly inoculated with SARS-CoV-2 Delta variant and contact ferrets in Groups A-D . Homologous SARS-CoV-2 virus neutralisation test (VNT) titres and ID Screen SARS-CoV-2 Double Antigen Multi-species ELISA (IDVet) from serum collected from experiment 2. Neutralisation titres are displayed as inhibition concentration 50% ($IC_{50}$) calculated using the Spearman-Karber method. The dashed horizontal line indicates the limit of detection for neutralisation equivalent to 1.41 $IC_{50}$. The dotted line indicates the positivity cut off ratio equivalent to 60%. Red cross indicates where the VNT could not be performed due to insufficient volume of sera available to test.
